# Non-Invasive miRNA Profiling for Differential Diagnosis and Prognostic Stratification of Testicular Germ Cell Tumors

**DOI:** 10.3390/genes15121649

**Published:** 2024-12-22

**Authors:** Panagiotis J. Vlachostergios, Konstantinos Evmorfopoulos, Ioannis Zachos, Konstantinos Dimitropoulos, Eleni Thodou, Maria Samara, Vassilios Tzortzis, Antonis Giakountis

**Affiliations:** 1 Department of Medical Oncology, IASO Thessalias General Hospital, 41500 Larissa, Greece; 2 Division of Hematology & Medical Oncology, Weill Cornell Medicine, New York, NY 10065, USA; 3 Department of Urology, Faculty of Medicine, University Hospital of Larissa, University of Thessaly, 41100 Larissa, Greece; 4 Department of Urology, Aberdeen Royal Infirmary, Aberdeen AB25 2ZN, UK; 5 Department of Pathology, Faculty of Medicine, School of Health Sciences, University of Thessaly, 41335 Larissa, Greece; 6 Department of Biochemistry & Biotechnology, School of Health Sciences, University of Thessaly, 41335 Larissa, Greece

**Keywords:** miRNA, testicular cancer, germ cell tumor, differential diagnosis, prognosis

## Abstract

Background/Objectives: Testicular germ cell tumors (TGCT) are common in young adult men and have high cure rates. Conventional serum tumor markers and imaging are not able to differentiate between histologic subtypes of the disease, which portend different prognoses and require distinct therapeutic strategies. Micro-RNAs (miRNAs) are small non-coding transcripts involved in the post-transcriptional regulation of gene expression, which have emerged as promising biomarkers in a variety of tumors. This study aimed to assess the potential of differentially expressed miRNAs in differential diagnosis and prognostication among TGCT patients with various histologic subtypes. Methods: Transcriptomic analysis of 134 patients from The Cancer Genome Atlas (TCGA)-TGCT database was conducted. miRNA differential expression analysis among seminomatous, embryonal carcinoma, mixed GCT, and teratoma was performed, followed by ROC curve analysis of the most significantly up- and downregulated miRNAs, respectively. Statistical associations of miRNA expression with AJCC stage were also investigated along with miRNA target network analysis and evaluation of miRNA detection in patients’ fluids. Results: Upregulation of seven miRNAs (hsa-mir-135a-1, hsa-mir-135a-2, hsa-mir-200a, hsa-mir-200b, hsa-mir-203b, hsa-mir-375, hsa-mir-582) and downregulation of seven additional miRNAs (hsa-mir-105-1, hsa-mir-105-2, hsa-mir-4433a, hsa-mir-548x, hsa-mir-5708, hsa-mir-6715a, hsa-mir-767) were identified. miRNAs displayed a high sensitivity/specificity of 0.94/1.0 (AUC = 0.98) for the upregulated and 0.97/0.94 (AUC = 0.96) for the downregulated signature. Deregulated expression of these miRNAs was significantly associated with AJCC stage and distant organ metastasis (*p* < 0.001), overall supporting their prognostic strength. Both signatures were detectable in body fluids, particularly urine. miRNA target network analysis supported the functional role of these miRNAs in the regulation of cancer-related processes such as cell proliferation via deregulation of pivotal oncogenes. Conclusions: These findings support the clinical value of two novel miRNA signatures in differential diagnosis and prognostic stratification of various histologic subtypes of TGCT, with potential treatment implications.

## 1. Introduction

Seminomatous and non-seminomatous germ cell tumors are not uncommon in young adults. Histopathological differential diagnosis is key to ensure selection of the most appropriate treatment for each patient [1]. Until present, serum tumor markers and imaging studies including computed tomography (CT), magnetic resonance (MR) imaging, and even photon emission tomography (PET)-CT have not been able to reliably differentiate between different histological subtypes [2]. The main differential diagnostic tools remain a panel of immunostains, to determine the presence and percent of embryonal carcinoma, yolk sac tumor, choriocarcinoma, or teratoma, as well as the presence of chromosome 12p amplification whenever differential includes non-TGCT diagnoses as well [1]. This becomes particularly important in cases where there is residual disease after initial chemotherapy, as these patients often need to undergo retroperitoneal lymph node dissection (RPLND), a challenging operation with high morbidity [3]. Thus, understanding the characteristics and management of residual masses after chemotherapy that may contain viable disease including seminoma, non-seminomatous GCT, or teratoma is key for optimizing treatment strategies and improving patient outcomes [4].

Additionally, seminomatous and non-seminomatous tumors have different prognosis. Almost three decades after the first International Germ Cell Consensus Classification (IGCCC) [5], prognostication using strictly clinicopathological parameters including primary site, degree of elevation of alpha-fetoprotein (AFP), human chorionic gonadotropin (HCG), and lactic dehydrogenase (LDH), and the presence of nonpulmonary visceral metastases (NPVM) remains the cornerstone of initial evaluation and has a pivotal role in the management of these patients [6].

In the era of precision medicine and personalized oncology, developing robust biomarkers that may identify distinct patient subpopulations who could derive the greatest benefit from contemporary and emerging therapies is extremely important. A better understanding of the molecular landscape of TGCTs has opened new avenues of research and opportunities for biomarker development [7,8].

Micro-RNAs (miRNAs) are small non-coding transcripts involved in the post-transcriptional regulation of gene expression [9]. They have emerged as promising biomarkers in a variety of diseases, including malignant tumors of various primaries [10]. To date, several miRNAs have been studied in TGCTs; however, there is no consensus on a definite miRNA signature that could serve as a tool for molecular stratification of patients with recurrent disease to different therapeutic strategies, emphasizing the necessity for further research [11]. This study aimed to assess the potential value of differentially expressed miRNAs across distinct histological TGCT subtypes as a clinical tool to assist with the differential diagnosis and prognosis of these patients.

Below, we present the results of a computational analysis that relies on publicly available TCGA data to evaluate miRNA expression in terms of differential diagnostic and prognostic performance across different histological subtypes of TGCTs. Our analysis provided a molecular signature consisting of seven upregulated miRNAs and seven downregulated ones that collectively outperform existing methods in differentiating between seminoma, embryonal carcinoma, mixed GCT, and teratoma. These miRNA signatures are of high prognostic relevance with respect to stage and distant metastases and their detection in various body fluids, particularly urine, could facilitate a non-invasive classification and prognostication of TGCTs.

## 2. Materials and Methods

### 2.1. TCGA Data Acquisition and Preprocessing

miRNA expression matrices were obtained from The Cancer Genome Atlas (TCGA) through the GDC portal (https://gdc.cancer.gov/, accessed on 1 May 2023), with the associated clinical information. More specifically, the expression of an initial miRNA pool consisting of 1881 was analyzed in 134 TCGT tumor specimens, stratified into 66 seminoma, 28 mixed germ cell, 26 embryonal carcinoma, and 14 teratoma tumors that represent the most common TGCT subtypes (Supplementary Table S1). The 1881 miRNAs were subjected to quality control filtering to eliminate transcripts with mean expression below the 25% percentile across all samples prior to downstream analysis. This resulted in a filtered miRNA matrix of 1386 transcripts corresponding to the 74% of the initial dataset. The same process was repeated to obtain expression data and clinical information for constructing the multi-cancer panel.

### 2.2. miRNA Differential Expression Analysis

Differential expression analysis was applied to the filtered dataset with the Bioconductor package edgeR (v4.4.0) (https://bioconductor.org/packages/release/bioc/html/edgeR.html, assessed on 18 July 2023). The following thresholds were applied: False Discovery Rate (FDR) < 0.05 and log2FC > 1 or < −1 for each of the following comparisons: (i) seminoma vs. non-seminoma tumors, (ii) seminoma vs. teratoma, (iii) seminoma vs. embryonal carcinoma, and (iv) seminoma vs. mixed germ cell tumors. Volcano plot illustrations were made with the Bioconductor package EnhancedVolcano in R (v1.22.0) (https://bioconductor.org/packages/release/bioc/html/EnhancedVolcano.html, assessed on 20 July 2023). For the heatmap analysis, z-score calculations of the log2 transformed expression values for the selected miRNAs were used. The TCGT tumor subtypes based on the associated clinical information were used as sample annotations for the heatmap. The Bioconductor ComplexHeatmap package was used for the final illustration. Venn plots were created in R.

### 2.3. ROC Analysis of Selected miRNA Signatures

Receiver Operating Curve (ROC) analysis, including calculations of AUC, sensitivity, and specificity was coupled to cut-off analysis with the GitHub package OptimalCutpoint (v1.1-5) as described in [12]. The following selection criteria were applied for constructing the miRNA signatures: *p*-value < 0.05 and AUC ≥ 0.95. All ROC plots were generated in R.

### 2.4. Violin Plots and Statistical Analysis of miRNA Expression Across Clinical Manifestations

Expression analysis for assessing the expression of the miRNA signatures against the TCGT patient clinical data was performed in R. More specifically, the following clinical manifestations were analyzed: (i) primary diagnosis, (ii) radiation treatment, (iii) AJCC metastasis (tumor M stage), and (iv) AJCC tumor stage, and the results were illustrated with violin plots using ggplot2(v.3.5.1) (https://ggplot2.tidyverse.org/, accessed on 13 September 2023) in R. ANOVA statistical test followed by multiple comparisons analysis was performed with SigmaPlot v11 (https://alfasoft.com/software/statistics-and-data-analysis/data-visulization/sigmaplot/, accessed on 7 July 2024).

### 2.5. Network and GO Analysis

miRNA target prediction and construction of the associated network was performed with miRNET [13], using the miRbase IDs of the selected miRNAs as input and performing the analysis with the miRTarBase v8 genes. Functional analysis of the miRNA targets was focused on GO BP, DisGeNET, and KEGG, and significance was assessed through hypergeometric enrichment. Dot plots for the illustration of the functional enrichment results were performed with ggplot2 in R (v.3.5.1) (https://ggplot2.tidyverse.org/, accessed on 14 July 2024), using the top 29 categories for clarity, ranked based on Gene ratio.

### 2.6. Multi-Cancer Diagnostic and Prognostic Evaluation

Multi-cancer ROC analysis was performed for each cancer type as described above. For survival analysis, the expression of the selected miRNAs along with the relevant clinical information (vital status) for each cancer type was used for performing Hazzard’s Ratio (HR) using SigmaPlot v11 (https://alfasoft.com/software/statistics-and-data-analysis/data-visulization/simaplot/, accessed on 23 September 2024). The HR results were illustrated through forest plot construction using the package forestploter https://cran.r-project.org/web/packages/forestploter/vignettes/forestploter-intro.html (accessed on 29 September 2024) in R (v1.1.2).

### 2.7. miRNA Expression in Circulating Body Fluids

For the evaluation of miRNA presence in body fluids, expression values (representing VSN levels) for selected miRNA panels were extracted from the Human miRNA Tissue Atlas database [14]. Dot plots were created with ggplot2 in R (v.3.5.1) (https://ggplot2.tidyverse.org/, accessed on 6 August 2024).

## 3. Results

### 3.1. miRNA Expression Analysis in TGCT Patient Tumor Samples

#### 3.1.1. miRNA Transcriptome Analysis

Differential expression analysis revealed extensive deregulation of miRNAs in patient tumor samples with testicular cancer. This analysis focused on comparisons between seminoma and non-seminoma tumors, since the latter are associated with poor prognosis, represent more aggressive forms of testicular cancer, and are accompanied by a significantly higher probability for metastasis compared to the former. A comparison between seminoma and mix germ cell tumors (MGCTs) revealed 308 differentially expressed miRNAs (abbreviated as DEMs thereof, Supplementary Table S2). These DEMs are further subdivided into 239 upregulated (representing 78% of the total) and 69 downregulated (representing 69% of the total) miRNAs. We also compared seminoma against embryonal carcinoma (EC) tumors and identified 256 DEMs. Again, the identified DEMs followed a similar pattern of deregulation with an over-representation of upregulated miRNAs (188 or 73% of total DEMs) compared to the downregulated ones (68 or 0.27% of total). Finally, a comparison of seminoma vs. teratoma (TE) tumors revealed 316 DEMs, with a slightly more balanced composition of 215 upregulated (68%) and 101 (32%) downregulated DEMs.

**Figure 1:**
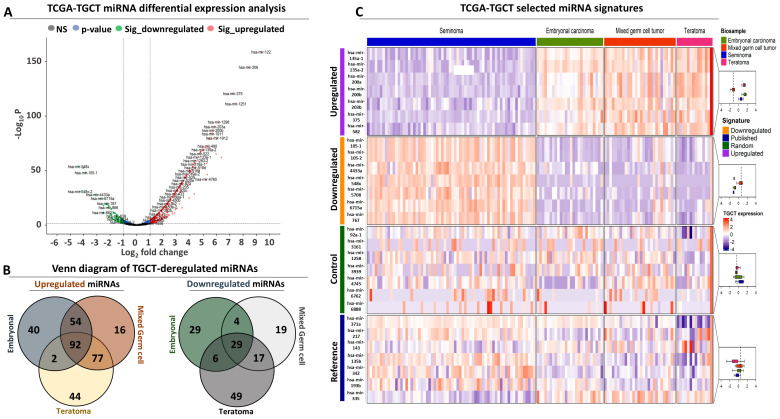
Differential expression analysis of miRNA expression in TCGT patients: (**A**) Volcano plot illustrating the statistically significant DEMs in seminoma against non-seminoma tumors. The latter include embryonal carcinoma (EC), teratoma (TE), and mixed germ cell tumors (MGCT). Red dots correspond to significantly upregulated miRNAs, green dots highlight the significantly downregulated miRNAs, and grey dots highlight the non-significant (NS) miRNAs. Blue dots refer to miRNAs that exceed the threshold of statistical significance (−log10 [*p* value] > 1.3, horizontal dashed line) but not the fold change threshold (<−1 or >1, vertical dashed lines). (**B**) Venn diagram comparing all DEMs for EC, TE, and MGCT against seminoma separately for the upregulated (left panel) and downregulated (right panel) miRNAs. (**C**) Heatmap illustrating the expression of the up- and downregulated miRNA signatures across all TGC tumors, separated according to their subtype (shown at the top). The reference and control miRNA signatures are also included for comparison. Boxplots on the right summarize the expression of each signature across subtypes.

With regards to deregulation strength, the average fold change in the upregulated DEMs in MGCTs was equal to 2.5 (±1.4), while the downregulated DEMs had an average fold change of −1.73 (±0.56) rpm. The upregulated miRNAs in EC tumors had an average fold change of 2.7 (±1.47), yet the downregulated pool had an average fold change of −1.95 (±1.07) suggesting a more variable composition compared to the downregulated DEMs in MGCTs. A similar conclusion can be drawn with regard to the deregulated miRNAs in TE tumors with average fold changes of 2.69 (±1.59) for the upregulated and −2.14 (±1.09) rpm for the downregulated DEMs, respectively. These observations are maintained when seminoma and non-seminoma tumors (composed of all the rest subtypes) are compared, revealing 244 upregulated DEMs with an average fold change of 2.57 (±1.42) and 56 downregulated DEMs with an average fold change of −1.72 (±0.78) rpm, respectively (Figure 1A, Supplementary Table S2). Taken together, these results suggest that the separation of testicular tumors into seminoma (with good prognosis) against each of the remaining subtypes (with intermediate or poor prognosis) is accompanied by extensive changes in terms of miRNA expression, with a potential for prognostic exploitation.

#### 3.1.2. Composing miRNA Signatures with a Prognostic Potential for TGCT

Having established separate pools of DEMs with significantly altered expression between seminoma and the remaining malignant subtypes of testicular cancer, we next focused on comparing these pools to reveal commonly affected DEMs in subtypes with poor prognosis. A comparison of the upregulated miRNAs in MGCTs, ECs, and TEs revealed 92 commonly affected DEMs between all subtypes (Figure 1B). MGC tumors shared most of their DEMs with the remaining two subtypes, retaining only 16 miRNAs (6.7% of all up-regulated miRNAs in MGCT) as uniquely upregulated in this subtype. A total of 169 MGCT-up-regulated miRNAs were also upregulated in the TE subtype, while EC tumors shared 146 miRNAs with MGCTs, possibly reflecting the mosaic of cellular types that compose the MGC tumors. Importantly, ECs and TEs shared 94 upregulated miRNAs while retaining 40 miRNAs (21% of all upregulated miRNAs) and 44 (20.4% of all upregulated miRNAs) as uniquely affected for each subtype, respectively.

In terms of downregulation, our analysis revealed 29 commonly affected miRNAs that were shared between the three subtypes. Similar to the observations for the upregulated DEMs, MGCTs had the lowest number of uniquely affected miRNAs (19 transcripts representing 27.5% of all downregulated miRNAs in this subtype), while sharing 33 downregulated miRNAs with ECs and 46 with TEs. In contrast, TEs had the highest number of uniquely downregulated miRNAs (49 or 48.5% of all TE-downregulated transcripts) and shared only 35 downregulated miRNAs with ECs, representing 34.6% of all TE-DEMs with reduced expression compared to seminoma levels (Figure 1B). Collectively, these results suggest that the upregulated miRNAs are more frequently shared between the three subtypes that are associated with poor prognosis, compared to the downregulated pool of miRNAs in which TE tumors maintain the largest number of miRNAs with uniquely affected expression only in this subtype.

Given the high proportion of commonly affected up- and downregulated miRNAs between the three subtypes, and since our primary aim was to isolate molecular signatures that distinguish between subtypes with good and bad prognoses, we selected two panels composed of fourteen DEMs (seven upregulated and seven downregulated) between seminoma and non-seminoma tumors, along with an equally sized collection of randomly selected miRNAs, which serve as a control signature. Heatmap analysis confirmed that only the selected miRNAs that compose our up- and downregulated signatures are strongly and uniformly deregulated between all non-seminoma compared to seminoma tumors (Figure 1C). To further challenge the performance of both miRNA panels, we included a third collection composed of seven miRNAs that were independently published as diagnostic/prognostic markers for TGCT, which we used as a reference signature. Despite the significant deregulation that characterized some of the reference miRNAs in separate comparisons between ECs, TEs, and MGCTs against seminoma tumors, only hsa-miR-335 was significantly deregulated in all TGCT subtypes with poor prognosis (Figure 1C, Supplementary Table S2). To conclude, these results suggest that our selected miRNA signatures are differentially expressed between TCGT subtypes with good vs. poor prognosis and retain their deregulated pattern of expression regardless of inter-patient heterogeneity.

### 3.2. Evaluating the miRNA Signatures in TCGT Patients

#### 3.2.1. Diagnostic and Prognostic Potential of the Upregulated Signature

**Figure 2:**
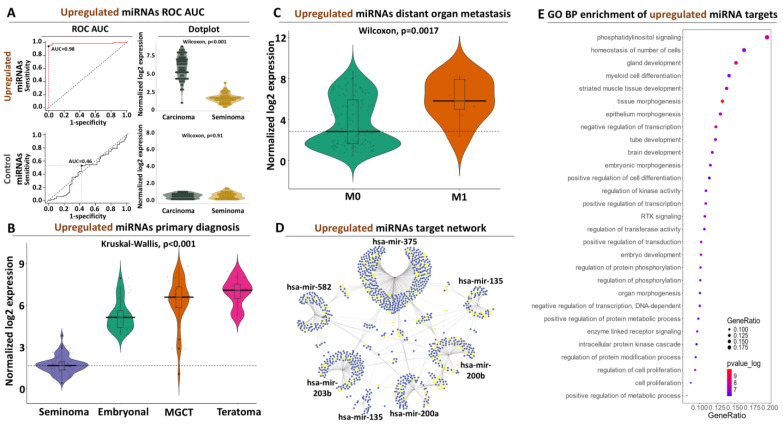
Characterization of the upregulated miRNA signature in TGCT patients: (**A**) ROC-AUC analysis for seminoma vs. non-seminoma discrimination (left panel) coupled to violin plot for contrasting miRNA expression in the same biosamples (right panel). The AUC performance of the control miRNAs along with their expression for the same comparison is shown at the bottom panels. (**B**) Violin plot illustrating the expression of the upregulated miRNAs across all TCGT subtypes. Horizontal line marks average miRNA expression in seminoma. (**C**) Same as (**B**) for ATCC M stage referring to distant organ metastasis. Horizontal line marks average miRNA expression in non-metastatic (M0) tumors. (**D**) miRNA target network analysis. Nodes correspond to the upregulated miRNAs, while blue edges correspond to their targets. Yellow dots highlight target genes that are associated with cell proliferation. (**E**) Functional enrichment analysis indicating biological processes that are significantly associated with the targets of the upregulated miRNAs.

Having established a panel of upregulated miRNAs, we next sought to thoroughly assess their potential in discriminating between seminoma and TGCT tumors with poor prognosis. ROC analysis confirmed that all miRNAs that compose this panel perform exceptionally well in separating seminoma and non-seminoma tumors (Supplementary Table S3). More specifically, each of the seven miRNAs from this panel is associated with AUC values that exceed 0.95, with *hsa-miR-135a-2* having the best performance (0.97) and *hsa-miR-375* the worst (0.95). When all miRNAs are combined, their collective AUC is increased even further (0.98), suggesting even higher diagnostic accuracy in contrast to the control miRNA signature (AUC = 0.46, Figure 2A) or the reference miRNAs (AUC = 0.24, Supplementary Figure S1A). The elevated AUC score of the upregulated panel was complemented with a significantly increased performance in terms of sensitivity (0.941) and specificity (1.0), highlighting again the enhanced diagnostic power of the upregulated signature.

Importantly, the same miRNAs retain their elevated discriminatory performance in separate comparisons between seminoma and each of the three poor prognosis subtypes (EC, TE, and MGCT, Supplementary Table S3), outperforming both the control (Supplementary Table S4) or the reference signatures (Supplementary Table S5) in all comparisons. These results align well with the significant and progressively increased expression of this miRNA panel in EC, MGCT, and TE tumors compared to seminomas (Figure 2B, Supplementary Table S6). Of note, the reference miRNA panel showed a progressively decreased pattern of expression (Supplementary Figure S1B), which was statistically significant predominantly between seminoma and teratoma tumors (Supplementary Table S6) in contrast to the control miRNAs that did not significantly alter their expression across the different subtypes as expected (Supplementary Figure S2A, Supplementary Table S6).

Encouraged by these observations, we expanded the analysis of our upregulated signature to other clinical manifestations, revealing significant differences between advanced M1 tumors compared to M0 neoplasms (Figure 2C, Supplementary Table S6), in contrast to the reference (Supplementary Figure S1C) or the control (Supplementary Figure S2B) signatures. We also observed significantly higher expression of the same miRNAs in the tumors of patients who did not receive any radiation treatment compared to treated patients (Supplementary Figure S3A, Supplementary Table S6). This time, the reference miRNAs significantly increased their expression in the radiated tumors (Supplementary Figure S1D) in contrast to the control signature (Supplementary Figure S2C). Interestingly, our upregulated miRNAs progressively increased their expression as the disease progressed from Stage I to Stage III, but not in a statistically significant manner (Supplementary Figure S3B), similar to the reference (Supplementary Figure S1E) or control (Supplementary Figure S2D) panels that were also not significantly altered between different tumor stages. Finally, we observed a progressive yet not significant decline in the expression of our upregulated miRNAs in older vs. younger patients (Supplementary Figure S3C). Variability in miRNA expression, especially in older patients, was the primary cause for lacking statistical significance, suggesting that a tighter selection of miRNAs from this panel could reveal significant associations. Importantly, we did not observe an equal decline nor any significant effects in the expression of the reference (Supplementary Figure S1F) or the control (Supplementary Figure S2E) panel. Taken together, these data support a significantly higher expression of our upregulated miRNA signature in non-radiated, EC, TE, or MGCT tumors with established metastasis in distant organs compared to seminoma neoplasms with good prognosis, underlying their crucial advantage in terms of prognostic utilization compared to previous reports.

#### 3.2.2. Target Network and Functional Analysis of the Upregulated miRNAs

Oncogenic miRNAs frequently increase their expression in tumors, facilitating initiation and/or progression of the disease through the degradation of downstream targets that govern cancer-related processes. To explore this possibility, we subjected our upregulated miRNA panel to miRNA::target prediction coupled with transcriptional network construction and functional analysis. As shown in Figure 2D, the upregulated miRNAs are predicted to target 1171 transcripts. Of them, 1051 are predicted to be targeted by a single miRNA, and 120 (10.2% of all) are collectively targeted by two or more miRNAs. In terms of complexity, *hsa-miR-375* is predicted to target the highest number of transcripts (477) while *hsa-miR-135a-2* only targets 36. In terms of combined targeting, *hsa-mir-375* shares its targets with all other miRNAs in the panel, adding extra weight to its regulatory implications with regard to TGCT progression. Moving beyond *hsa-mir-375*, several common targets are also shared between *hsa-mir-200a* and *b*, which also retain a separate pool of commonly affected transcripts with *hsa-mir-375*, highlighting these three regulatory small RNAs as important regulatory nodes within our upregulated signature (Figure 2D).

miRNA targeting by itself does not imply any biological impact, unless the affected transcripts govern critical physiological or pathological processes. To explore this possibility, we subjected the predicted target mRNAs of our upregulated signature to functional analysis. This strategy revealed that several developmental and cancer-related processes, including but not limited to cell proliferation, PI3k signaling, embryonic morphogenesis, and apoptosis, are associated with the predicted targets of our miRNAs (Figure 2E, Supplementary Table S7). What is more, the 166 transcripts (14% of all targets) that are associated with cell proliferation, belong to mRNAs that are commonly targeted by two or more miRNAs of this panel, suggesting the existence of functional redundancy in this crucial cellular process within our upregulated signature (Figure 2D). Beyond molecular function, disease pathway analysis revealed the enrichment of various forms of cancer that extend beyond TGCT across various organ primaries, highlighting the central regulatory role of these miRNAs in the disease (Supplementary Table S7). Overall, these data provide a mechanistic basis behind the collective upregulation of this miRNA signature in tumors with poor prognosis, which functionally underlines their diagnostic and prognostic potency.

#### 3.2.3. Diagnostic and Prognostic Potential of the Downregulated Signature

**Figure 3:**
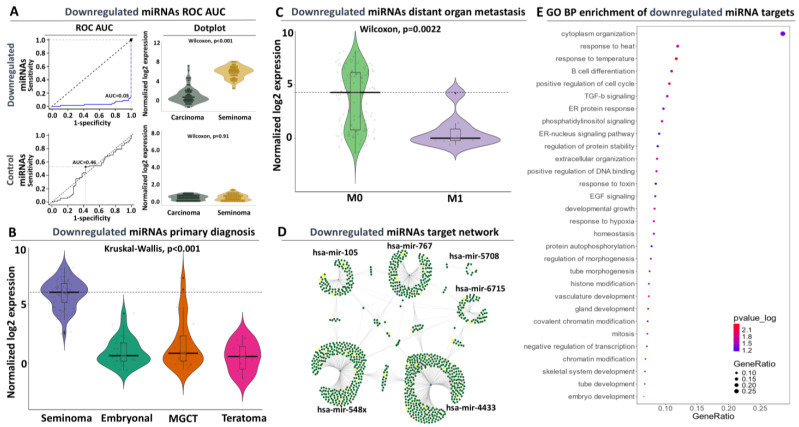
Evaluation of the downregulated miRNA signature in TGCT patients: (**A**) ROC-AUC analysis for seminoma vs. non-seminoma discrimination (left panel) coupled to violin plot for contrasting miRNA expression in the same biosamples (right panel). The AUC performance of the control miRNAs along with their expression for the same comparison is shown at the bottom panels. (**B**) Violin plot illustrating the expression of the downregulated miRNAs across all TCGT subtypes. Horizontal line marks average miRNA expression in seminoma. (**C**) Same as (**B**) for ATCC M stage referring to distant organ metastasis. Horizontal line marks average miRNA expression in non-metastatic (M0) tumors. (**D**) miRNA target network analysis. Nodes correspond to the downregulated miRNAs, while green edges correspond to their targets. Yellow dots highlight target genes that are associated with cell proliferation. (**E**) Functional enrichment analysis indicating biological processes that are significantly associated with the targets of the downregulated miRNAs.

With regard to our downregulated miRNA panel and similar to the upregulated signature observations, ROC-AUC analysis revealed elevated discriminatory power, with a cumulative AUC performance reaching 99.9 (for seminoma detection or 0.03 for non-seminoma tumors, Figure 3A, Supplementary Table S8). This result complemented the statistical differences that we observed in the expression of these miRNAs across all TCGT subtypes (Figure 3B, Supplementary Table S6). Equally high was the performance of this signature in terms of sensitivity (0.97) and specificity (0.91). In terms of individual miRNAs, we observed high and extremely compact AUC scores (above 0.98 for seminoma detection) for all miRNAs. Importantly, the same panel retained its enhanced detection potency for seminoma against each of the remaining TGCT subtypes (EC, TE, and MGCT, Supplementary Table S8), confirming the elevated discriminatory power for seminoma tumors that are associated with good prognosis. Taken together, these observations complement the results of our upregulated signature, offering two separate miRNA panels for specific and highly accurate separation of TGC tumors with a good or bad prognosis.

Moving beyond subtype discrimination, we observed the exact opposite trend compared to the upregulated panel in terms of miRNA expression with regard to distant organ metastasis, marked by a statistically significant decrease in M1 tumors, which independently confirms the elevated presence of this panel in non-metastatic tumors (Figure 3C, Supplementary Table S6). This significantly decreased expression persisted also in non-irradiated tumors compared to those from patients who received radiation treatment (Supplementary Figure S3D, Supplementary Table S6). Interestingly, in contrast to our upregulated panel that lacked significance, we observed a statistically significant decline in the expression of our second panel between Stage I and Stage II–III tumors, adding extra weight to their prognostic impact (Supplementary Figure S3E, Supplementary Table S6). Finally, we observed a progressive increase in the expression of these miRNAs with patient age; however, similar to the observations for the upregulated miRNAs, this altered expression was not significant (Supplementary Figure S3F, Supplementary Table S6). Collectively, our differential expression and ROC analysis yielded a second miRNA panel with expression properties that allow highly accurate discrimination of early-stage TCG tumors that respond well to radiation treatment.

#### 3.2.4. Target Network and Functional Analysis of the Downregulated miRNAs

Target network analysis predicted 1445 transcripts as targets of our downregulated miRNA panel. Of them, 1298 are uniquely targeted and 147 (corresponding to 10% of all targets) are targeted by two or more miRNAs (Figure 3D). This analysis also revealed two central miRNA regulatory nodes that are mediated through *hsa-mir-4433* and *hsa-mir-548x*. More specifically, *miR-4433* targets 373 and *miR-548x* targets 293 transcripts. Among the remaining members of this panel, *hsa-mir-767*, *hsa-mir-105*, and *hsa-mir-6715* target progressively smaller pools of 187, 107, and 80 transcripts, respectively, while *hsa-mir-5708* is very selective and targets only 13 transcripts. With regard to commonly affected targets, *hsa-mir-548x* and *4433* both retain a pool of 17 common targets; however, each miRNA shares independent pools of commonly targeted transcripts with each of the remaining miRNAs within this signature. These observations suggest that the selected miRNAs of our downregulated signature maintain high levels of expression in seminoma tumors forming a network of regulatory interactions that independently or collectively dictate the expression of hundreds of downstream target genes.

As a next step, we subjected these target genes to functional analysis for detecting associations with deregulated cellular processes and diseases. Indeed, we observed significant enrichment of physiological and cancer-related functions, which include but are not limited to gland formation, tube and vasculature morphogenesis, cell proliferation, and TGF-b or EGF signaling (Figure 3E). Of particular interest are the commonly affected targets between hsa-mir-767 and 548x, which show significant associations with histone remodeling and embryo development. In terms of disease associations beyond TGCT and in agreement with our previous observations, we observed significant enrichment of other types of cancer, which primarily referred to prostate and non-small cell lung cancer (Supplementary Table S9). In conclusion, analysis of the miRNAs that compose the downregulated panel revealed a network of regulatory interactions with mRNA targets that governs an amalgamation of developmental and cancer-related processes, providing functional support for their persistent upregulation in TGC tumors with good prognosis. The elevated specificity and sensitivity of this second signature for seminoma tumors complement the discriminatory properties of our upregulated panel for TGC tumors with poor prognosis, offering the opportunity of combining both miRNA pools into a dual panel for binary detection of TGCT.

### 3.3. Prognostic and Diagnostic Evaluation Beyond TGCT

#### 3.3.1. Evaluation of miRNA Signatures Through Multi-Cancer ROC Analysis

A fundamental advantage of miRNA panels compared to traditional diagnostic and prognostic approaches, is their universal presence in most, if not all, tissues and/or cancer types. This offers the unique opportunity of directly testing the performance of these transcripts as biomarkers in multi-cancer panels following the detection of their deregulated expression with next-generation sequencing. Following this approach, we subjected both miRNA signatures to ROC-AUC analysis designed to evaluate their diagnostic power in 20 different forms of cancer. To challenge their performance even more, we included the reference signature as well as the control miRNA panel, which served as a control in the analysis.

**Figure 4:**
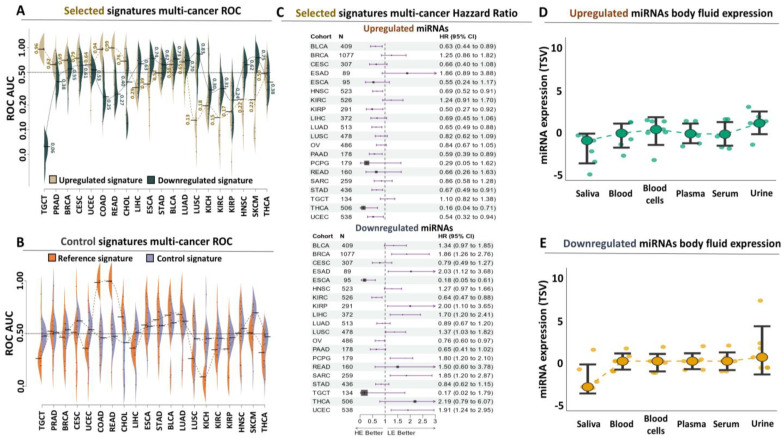
Assessment of the diagnostic and prognostic performance of both signatures in a multi-cancer panel: (**A**) Beanplots summarizing ROC-AUC performance of the upregulated (brown) or downregulated (green) signature across a multi-cancer panel. Tumor abbreviations are available here (https://gdc.cancer.gov/resources-tcga-users/tcga-code-tables/tcga-study-abbreviations, accessed on 1 May 2023). Numbers indicate average AUC performance for each cancer type. Horizontal line marks absence of diagnostic power (0.5). (**B**) Same as (**A**) for the reference (orange) or control (blue) signature. (**C**) Forest plot summarizing the results of the Hazzard’s Ratio (HR) analysis of the upregulated (upper panel) or downregulated (lower panel) signature for overall survival (OS) across all cancer types. The black vertical line marks the reference HR (=1) numbers on the right indicate average HR along with confidence intervals for each form of the disease. (**D**) Expression profiling of the upregulated miRNAs in body fluids of cancer patients. (**E**) Expression profiling of the downregulated miRNAs in body fluids of cancer patients.

Our multi-cancer approach confirmed the exceptionally high discriminatory properties of both signatures for TGCT (Figure 4A). More specifically, TGCT was the only type of cancer in which both signatures were simultaneously associated with AUC values above 0.95 for poor prognosis (non-seminoma, upregulated panel) or good prognosis (seminoma, downregulated panel). In all other forms of the disease, the same panels, either independently or in combination, were associated with lower AUC performance in terms of cancerous or paracancerous tissue detection, with the exception of colorectal cancer in which the upregulated miRNAs performed equally well with TCGT (Supplementary Table S10). However, in the same cancer, the downregulated panel was associated with worse AUC scores, suggesting poor diagnostic performance in discriminating paracancerous epithelium from colonic neoplasia. In all other cases, the average AUC score between the two panels was almost identical and approached the median (0.5), a trend which we interestingly observed also for reference compared to the control miRNAs (Figure 4B, Supplementary Table S10). Overall, our multi-cancer ROC analysis suggests that the combination of our proposed miRNA signatures is highly specific for TGC diagnosis compared to all other forms of the disease.

#### 3.3.2. Assessing the Prognostic Power of miRNA Signatures Through Multi-Cancer Hazzard Ratio Analysis

Having ensured the excelling performance of both signatures for TGCT with multi-cancer ROC, we decided to challenge them even further, this time through evaluation of their prognostic significance in terms of overall survival (OS) across the same multi-cancer panel. With regard to the upregulated miRNA panel, Hazzard’s Ratio (HR) analysis revealed a score of 1.10 (with Confidence Interval or CI between 0.82 and 1.38, Figure 4C, Supplementary Table S11). However, this was the fourth top-ranking performance for this signature, since esophageal, mammary, and certain kidney tumors were associated with even higher HRs (1.86, 1.25, and 1.25), respectively. Of note, we did observe a high variability of esophageal cancer, suggesting differences in terms of prognostic power between the miRNAs that compose the upregulated panel. Importantly, in most of the other forms of the disease, the HR score ranged below yet close to one, suggesting that TGCT remains in the top 20% of all cancers in terms of ranking based on the prognostic power of these miRNAs for OS.

We complemented this analysis with the results of our downregulated miRNAs in the same multi-cancer panel (Figure 4C, Supplementary Table S11). This second signature performed even better as a prognostic biomarker for OS, since TGCT was associated with the lowest HR score (0.17 with CI between 0.02 and 1.79). Despite this low HR score, we should also point out the observed variability between the different miRNAs, which was not unique for TGCT but was observed for most of the remaining cancers and could be attributed in part to inter-patient variability. Interestingly esophageal and kidney tumors were again among the top-performing forms of the disease, while, interestingly, most of the remaining tumors were associated with HR scores that ranged above one, implying that the downregulated miRNA panel could serve as a risk factor for shorting of survival time (and therefore poor prognosis) in certain forms of the disease. This observation highlights even more the specific role of these miRNAs as prognostic biomarkers for TGCT, and along with the detection of both the upregulated (Figure 4D) and downregulated (Figure 4E) miRNAs in body fluids (predominantly urine) of cancer patients emphasizes their role as a novel and highly specific method for non-invasive detection and prognosis of TGCT.

## 4. Discussion

While TGCT remains a curable disease, even in advanced stages, a proportion of patients will experience recurrence or residual disease after initial orchiectomy or/and cisplatin-based chemotherapy and will require additional therapeutic manipulations [3,11]. For these patients, differentiating histological subtypes of viable disease including teratoma, as well as prognostication, still represents a complex challenge inadequately addressed by contemporary histological, clinical, and biochemical parameters [15]. Stratification of patients in subgroups that could benefit from individual intensification or de-intensification of therapy, including RPLND, additional chemotherapy, or observation is key in order to improve their outcome. A plethora of emerging molecular biomarkers, including microRNAs, gene expression profiles, and immune-related biomarkers are currently under investigation in TGCTs [8].

Within the last decade, the *microRNA-371a-3p* (*miR371*) has yielded the most promising results as a potential biomarker in TGCT. Individual studies and meta-analyses demonstrated a sensitivity and specificity of approximately 90%, which outperforms those of classical serum tumor markers [16,17]. Circulating *miR-371a-3p* levels correlate with primary tumor mass, clinical stage, and IGCCC risk groups, supporting its potential incorporation into the clinical armamentarium of diagnostic and prognostic tools for clinical decision making in patients with TGCTs [18]. Furthermore, serum *miR-371a-3p* was shown to predict viable germ cell tumors in residual masses after chemotherapy with a high discriminative capacity (AUC of 0.87) [19]. Nevertheless, in certain clinical scenarios such as pre-chemotherapy non-seminomatous tumors, as well as in the post-chemotherapy setting, measuring *miR-371a-3p* levels is of limited utility according to the SWENOTECA-MIR study [20]. Additionally, teratoma does not express *miR-371a-3p* [21], and median relative expression of pre-RPLND serum miR-371a-3p is not significantly higher in seminomas compared to benign histology [22], suggesting that miRNA biomarkers may require further refinement in these settings.

The performance of our novel up- and downregulated miRNA signatures in TCGA-TGCT data was superior to the ones reported for *miR-371a-3p*. This lower diagnostic power and accuracy of the latter and other published miRNAs could be at least partially due to their original detection via RT-qPCR in serum, while TCGA-TGCT data are generated through miRNA sequencing in surgical pathology specimens. However, our selected miRNAs are also present in whole blood, serum, plasma, and urine. Additionally, the performance of our miRNA signatures, in terms of differential expression and prognosis across different TGCT histological subtype tissues, outperforms a large number of solid tumor types, properties that were not tested for *miR-371a-3p*.

Other miRNAs such as *miR-372-3p* [23,24], *miR-21*, *miR-29a*, and *miR-106b* [25] have been tested in various small-size studies with sensitivities and specificities of around 90%. Notably, none have proven to be able to distinguish teratoma from nonmalignant necrotic/fibrotic tissues or nonviable tumors in patients with NSGCT undergoing post-chemotherapy RPLND [26].

There are sparse data on potentially prognostic miRNAs in TGCTs. The expression of *miR-371a-3p* together with *miR-371a-5p* and *miR-29c-5p* was suggested to be predictive of the metastatic potential in seminomas, however only in ten patients [27]. High serum levels of *miR-371a-3p*, *miR-373-3p*, and *miR-367-3p* were reported to predict recurrence and worse outcomes in metastatic TGCT patients [28]. Other miRNAs including *miR-512-3p*, *miR-515*, *miR-517*, *miR-518*, *miR-525*, *miR-99a*, *miR-100*, and *miR-145* were found to promote cisplatin-resistance, which is associated with poor prognosis, however, none with high yield to support a meaningful clinical utility [29]. In contrast, our panel of up- and downregulated miRNA signatures were clearly and significantly associated with stage and distant metastases, supporting its strong prognostic utility both within TGCT tumors and among other tumor types. Overall, compared to existing evidence on miRNA signatures for TGCT, our up- and downregulated panel is not only entirely new, without any overlap with previously identified ones, but also has higher sensitivity and specificity for differential diagnosis of different TGCT histological subtypes and prognosis of these patients, respectively.

With respect to target prediction and function, our miRNA network and GO analysis suggest that both signatures regulate target networks that, in turn, govern critical cancer-related aspects of TGCT biology, such as PI3K signaling, and cytoplasm organization. For example, *miR-375* is inversely associated with THBS2 thatencodes thrombospondin, which is involved in extracellular matrix function that activates PI3Ks [30]. *miR-200a* and *miR-200b* promote phosphorylation of PI3K and Akt through targeted inhibition of *PTEN* gene expression [31]. Additionally, *miR-582* inhibits forkhead box protein O3 (FOXO3), resulting in upregulation of the PI3K/AKT/Snail signaling pathway [32]. Conversely, *miR-203* acts as a negative regulator of the PI3K pathway via direct suppression of CAV1 [33]. Aberrantly binding miRNAs such as *miR-548* can bind to nuclear hormone receptor genes resulting in the regulation of several downstream signaling pathways [34]. Taken together, this evidence not only supports our GO results regarding the critical function of our miRNA signatures in TGCTs but also provides a functional basis for the observed differential expression of these selected miRNAs, as well as a prognosis among different TGCT histological subtypes, paving the way for their future experimental validation with in vitro models.

A major limitation of our study refers to its retrospective computational design that solely exploits the TCGA patient cohort without in vitro validation. Our focus on the TCGA panel was based on detailed clinical information along with the use of deep sequencing for assessing miRNA expression in a large group of patients. Moreover, it provided us with the opportunity to compare our results across all other cancer types, representing a strong hypothesis-generating analysis that may pave the way for further validation in independent datasets or in vitro and in vivo investigations. These results could substantially help evaluate the full potential of both miRNA signatures for differential diagnosis and prognosis among patients with distinct histological TGCT subtypes, as well as elucidate the molecular mechanisms accounting for their function in TGCTs.

More importantly, differentiating between various subtypes of TGCTs using non-invasive methods such as measurement in urine might be particularly useful for facilitating patient stratification to different treatment options, including RPLND vs. observation in residual tumors.

## 5. Conclusions

This computational study evaluated two miRNA signatures with highly predictive power for TGCT differential diagnosis and prognosis. The significance of our findings lies in both the enhanced sensitivity and specificity of the reported signatures and their abundance in the body fluids of testicular cancer patients. Furthermore, this study provides bioinformatic evidence regarding the molecular mechanisms that support the functional role and prognostic potential of these miRNAs. Currently, we seek to experimentally validate the diagnostic and prognostic accuracy of our miRNA signatures in biopsies and body fluids of TGCT patients as part of a clinical trial that we are designing as a follow-up study. Provided their proper clinical validation, we propose both miRNA signatures as novel diagnostic and prognostic biomarker TGCT histological subtypes in a highly accurate and non-invasive manner. Collectively, our study supports the value of specific miRNA signatures as non-invasive molecular biomarkers for distinguishing between patients with different TGCT histological subtypes that have a distinct prognosis and require tailored treatment decision making.

## Data Availability

The original contributions presented in this study are included in the article/Supplementary Material. Further inquiries can be directed to the corresponding author.

## Supplementary Materials

The following supporting information can be downloaded at: https://www.mdpi.com/article/10.3390/genes15121649/s1, Figure S1: Evaluation of the reference miRNA signature in TGCT patients; Figure S2: Illustrating the expression of the control miRNA signature in TGCT patients; Figure S3: Profiling of the up- and downregulated miRNAs in clinical manifestations of TGCT patients; Table S1: Composition of the TCGA TGCT patient cohort; Table S2: Differential expression and statistical analysis of miRNAs in the TCGA TGCT dataset; Table S3: ROC and cut-off statistical analysis of the upregulated miRNA signature in TCGA TGCT patients; Table S4: ROC and cut-off statistical analysis of the control miRNA signature in TCGA TCGT patients; Table S5: ROC and cut-off statistical analysis of the reference miRNA signature in TCGA TCGT patients; Table S6: Statistical analysis of selected miRNAs expression in TCGA TGCT clinical cohorts; Table S7: Functional enrichment analysis of upregulated miRNA signature; Table S8: ROC and cut-off statistical analysis of the downregulated miRNA signature in TCGA TCGT patients; Table S9: Functional enrichment analysis of upregulated miRNA signature; Table S10: Multi-cancer ROC AUC analysis; Table S11: Multi-cancer Hazzard Ratio analysis.
