# Supplementary material for: Non-Invasive miRNA Profiling for Differential Diagnosis and Prognostic Stratification of Testicular Germ Cell Tumors
Source: Genes (Basel). 2024 Dec 22;15(12):1649. doi: 10.3390/genes15121649 (PMC11728082; doi:10.3390/genes15121649)
Supplement: Supplementary file 1 [file genes-15-01649-s001.zip › Supplementary Figures with legends.pdf]

Figure legends

Supplementary Figure 1 (related to Figures 2 and 3)

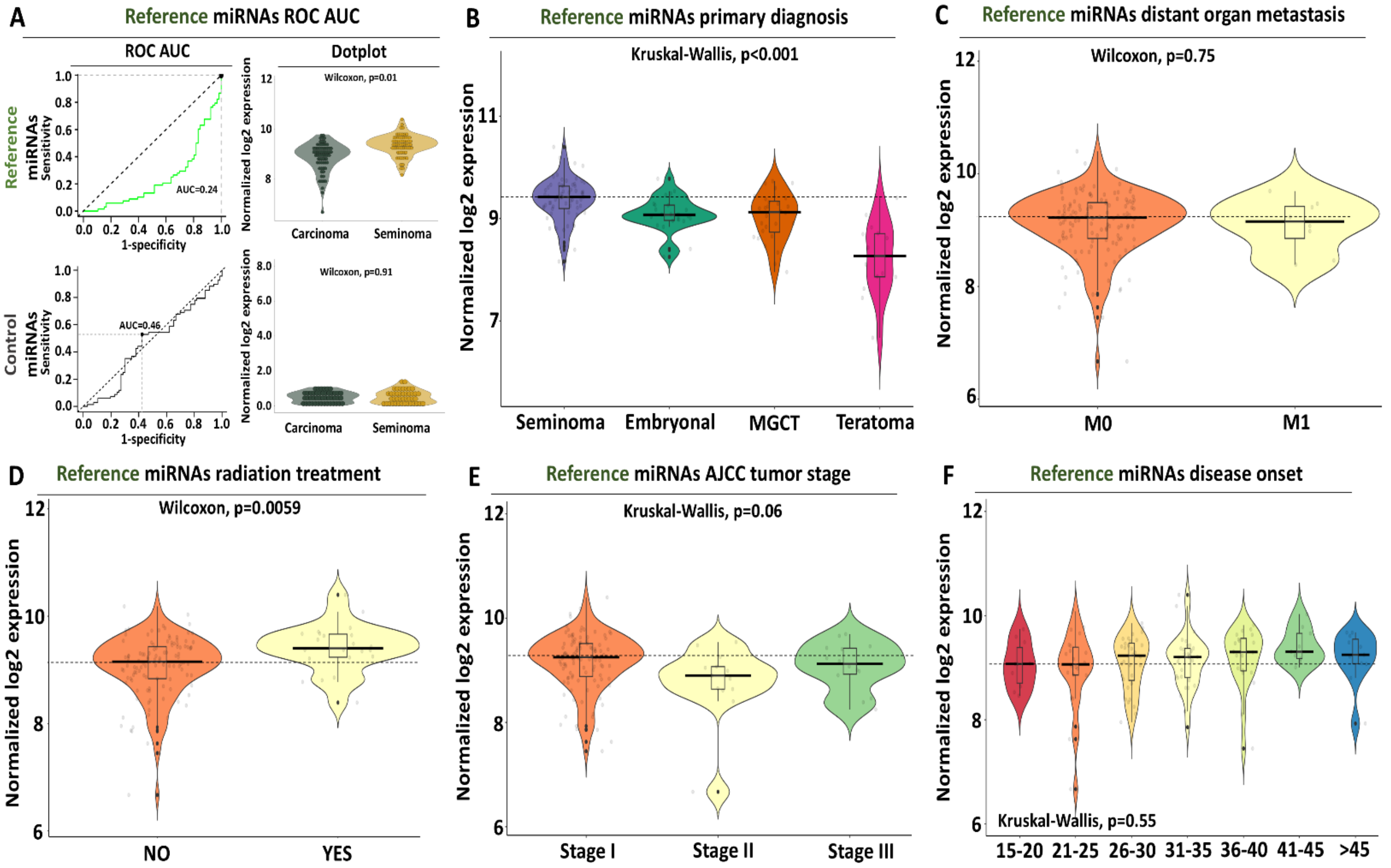

Supplementary Figure 1 Evaluation of the reference miRNA signature in TGCT patients. A) ROC-AUC analysis for seminoma vs non-seminoma discrimination (left panel) coupled to violin plot for contrasting the expression of the reference miRNA in the same biosamples (right panel). The AUC performance of the control miRNAs is shown at the bottom panels **B)** Violin plot illustrating the expression of the reference miRNAs across all TCGT subtypes. Horizontal line marks average miRNA expression in seminoma. **C)** Same as (**B**) for ATCC M stage referring to distant organ metastasis. Horizontal line marks average miRNA expression in M0 tumors. **D)** Same as (**B**) for radiation treatment. Horizontal line marks average miRNA expression in non-radiated tumors. **E)** Same as (**B**) for ATCC tumor stage. Horizontal line marks average miRNA expression in early (stage I) tumors. **F)** Same as (**B**) for disease onset. Horizontal line marks average miRNA expression in young patients.

Supplementary Figure 2 (related to Figures 2 and 3)

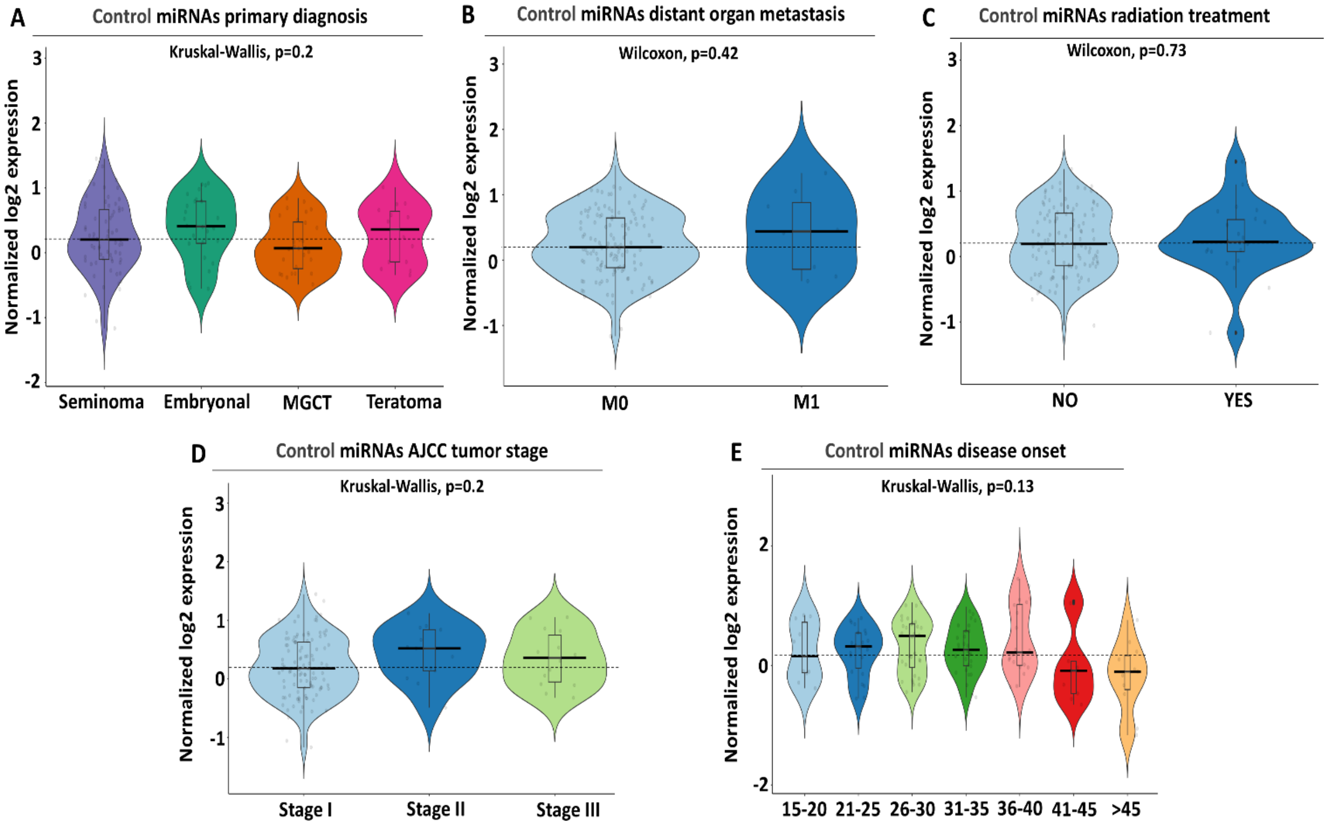

Supplementary Figure 2 Illustrating the expression of the control miRNA signature in TGCT patients. A) Violin plot illustrating the expression of the control miRNAs across all TCGT subtypes. Horizontal line marks average miRNA expression in seminoma. **B)** Same as (**A**) for ATCC M stage referring to distant organ metastasis. Horizontal line marks average miRNA expression in M0 tumors. **C)** Same as (**A**) for radiation treatment. Horizontal line marks average miRNA expression in non-radiated tumors. **D)** Same as (**A**) for ATCC tumor stage. Horizontal line marks average miRNA expression in early (stage I) tumors. **E)** Same as (**A**) for disease onset. Horizontal line marks average miRNA expression in young patients.

Supplementary Figure 3 (related to Figures 2 and 3)

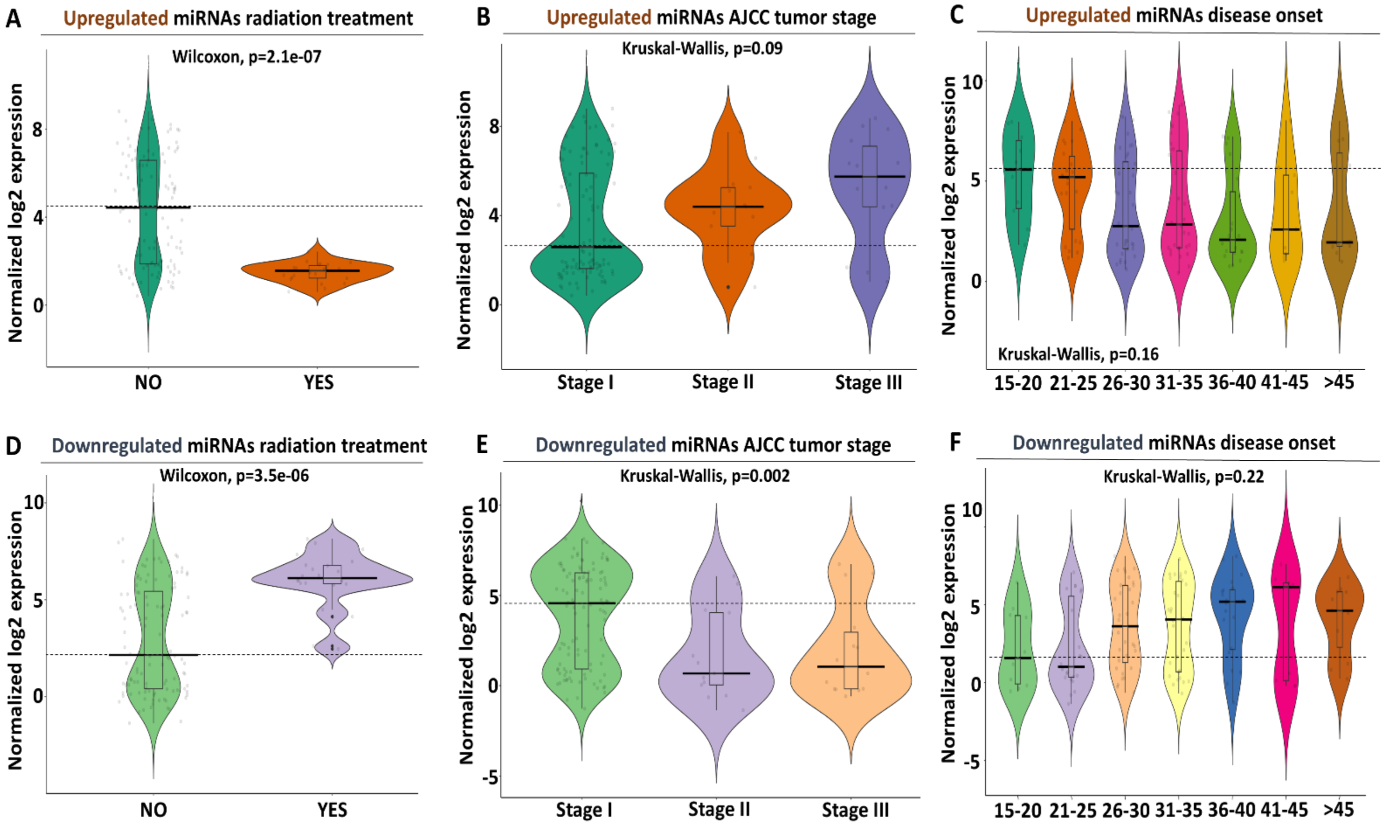

Supplementary Figure 3 Profiling of the up- and downregulated miRNAs in clinical manifestations of TGCT patients. A) Violin plot illustrating the expression of the upregulated miRNAs in radiated and non-radiated TGCT tumors. Horizontal line marks average miRNA expression in non-radiated tumors. **B)** Violin plot illustrating the expression of the upregulated miRNAs across tumor stages. Horizontal line marks miRNA expression in early (stage I) tumors. **C)** Violin plot illustrating the expression of the upregulated miRNAs for disease onset. Horizontal line marks average miRNA expression in young patients. **D)** Same as (**A**) for the downregulated signature. **E)** Same as (**B**) for the downregulated signature. **E)** Same as (**F**) for the downregulated signature.
