# Supplementary material for: Non-Invasive miRNA Profiling for Differential Diagnosis and Prognostic Stratification of Testicular Germ Cell Tumors
Source: Genes (Basel). 2024 Dec 22;15(12):1649. doi: 10.3390/genes15121649 (PMC11728082; doi:10.3390/genes15121649)

Supplementary Figure 1 (related to Figures 2 and 3)

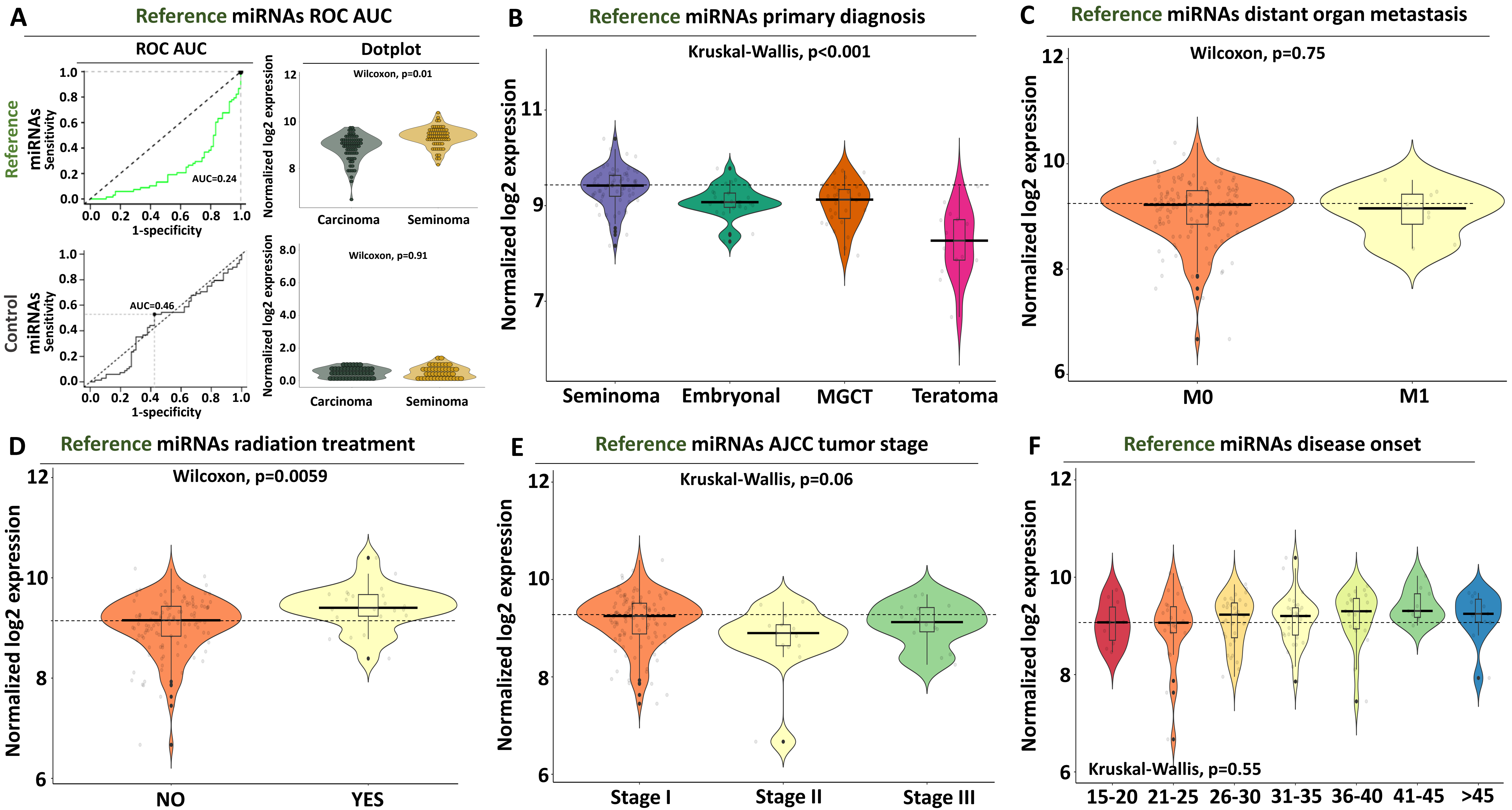

Supplementary Figure 2 (related to Figures 2 and 3)

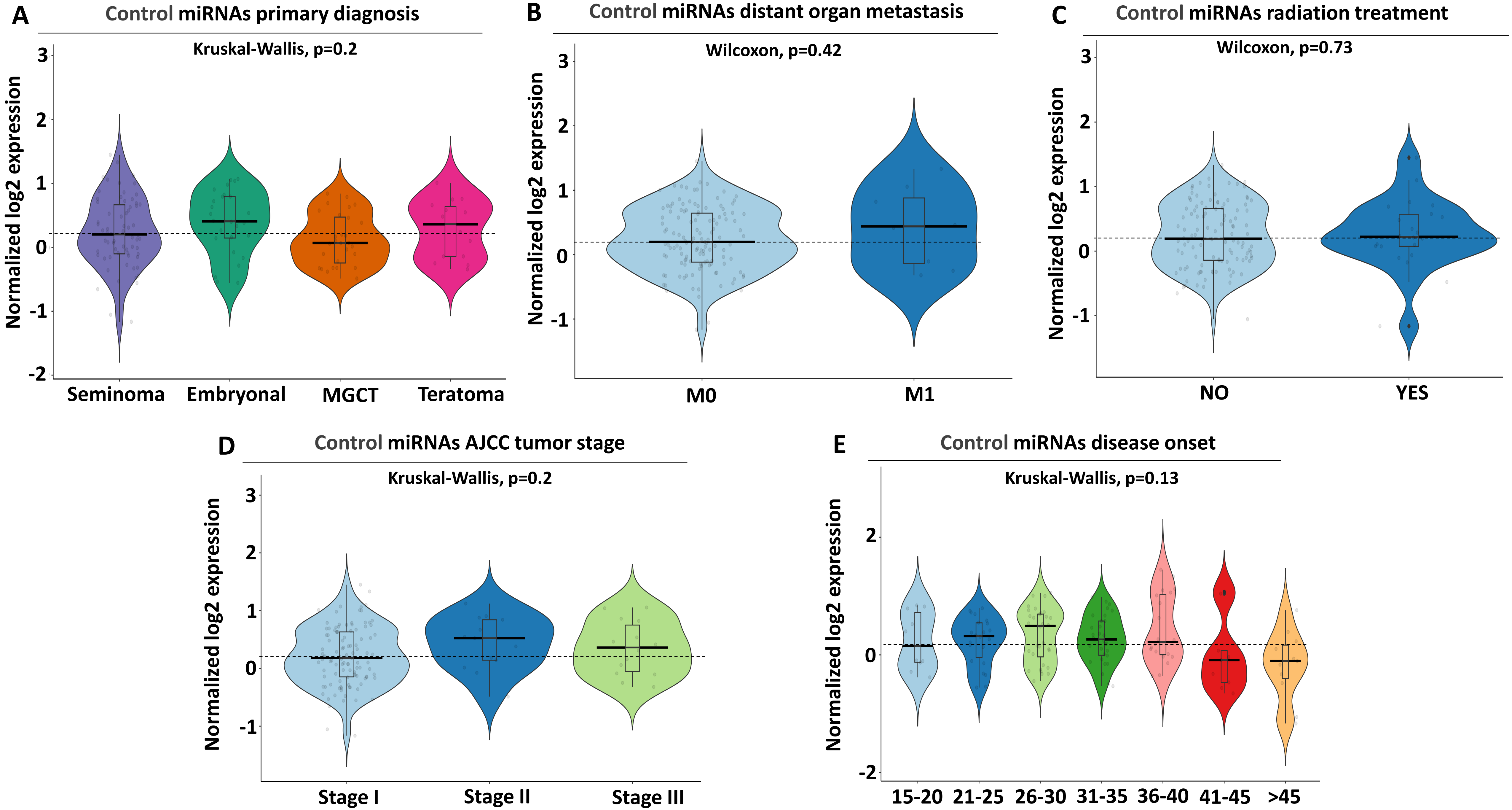

Supplementary Figure 3 (related to Figures 2 and 3)

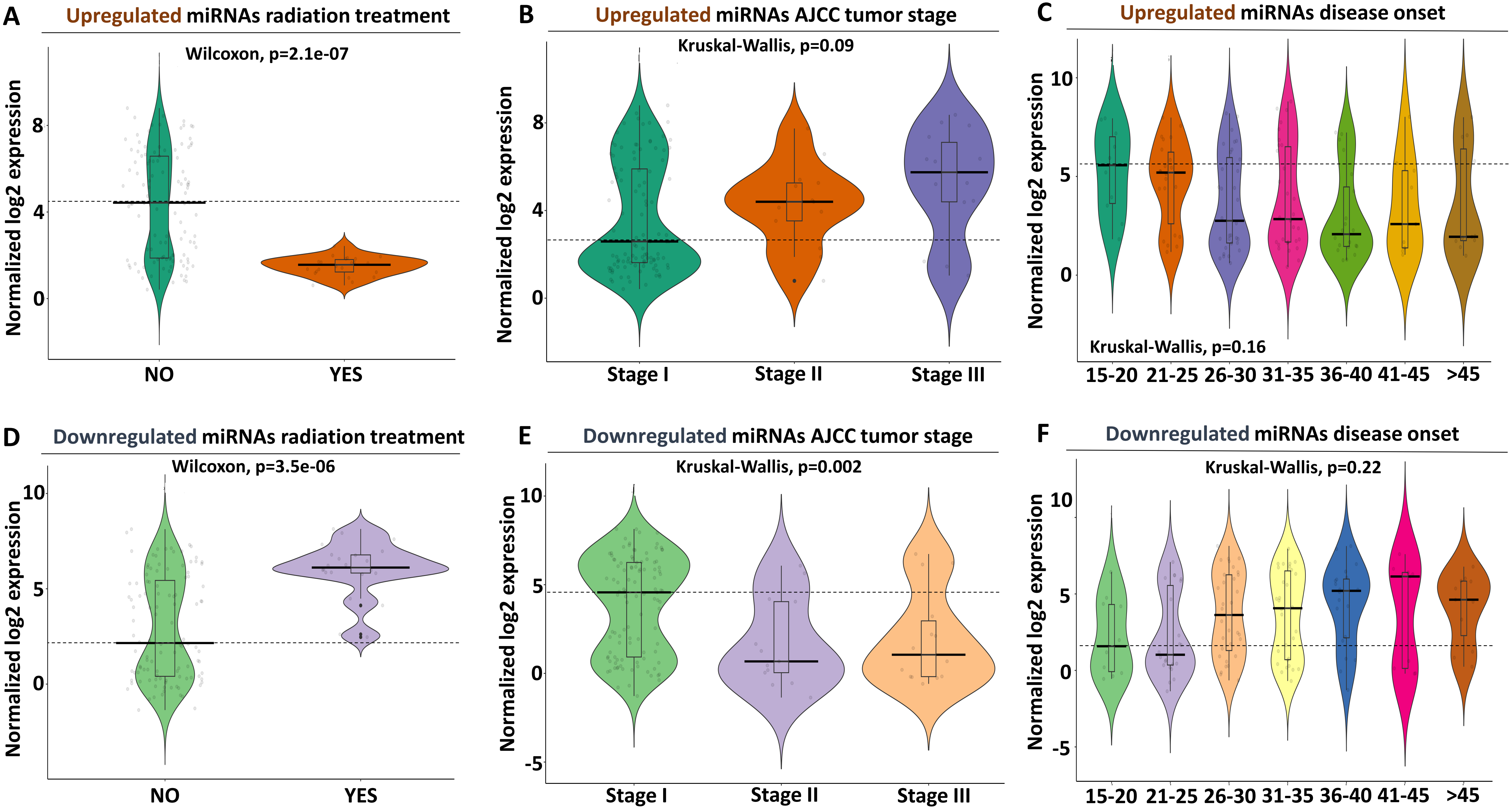

Supplement: Supplementary file 1 [file genes-15-01649-s001.zip › Supplementary Figures.pdf]
