# Supplementary figures and images for: Non-Invasive miRNA Profiling for Differential Diagnosis and Prognostic Stratification of Testicular Germ Cell Tumors
Source: Genes (Basel). 2024 Dec 22;15(12):1649. doi: 10.3390/genes15121649 (PMC11728082; doi:10.3390/genes15121649)

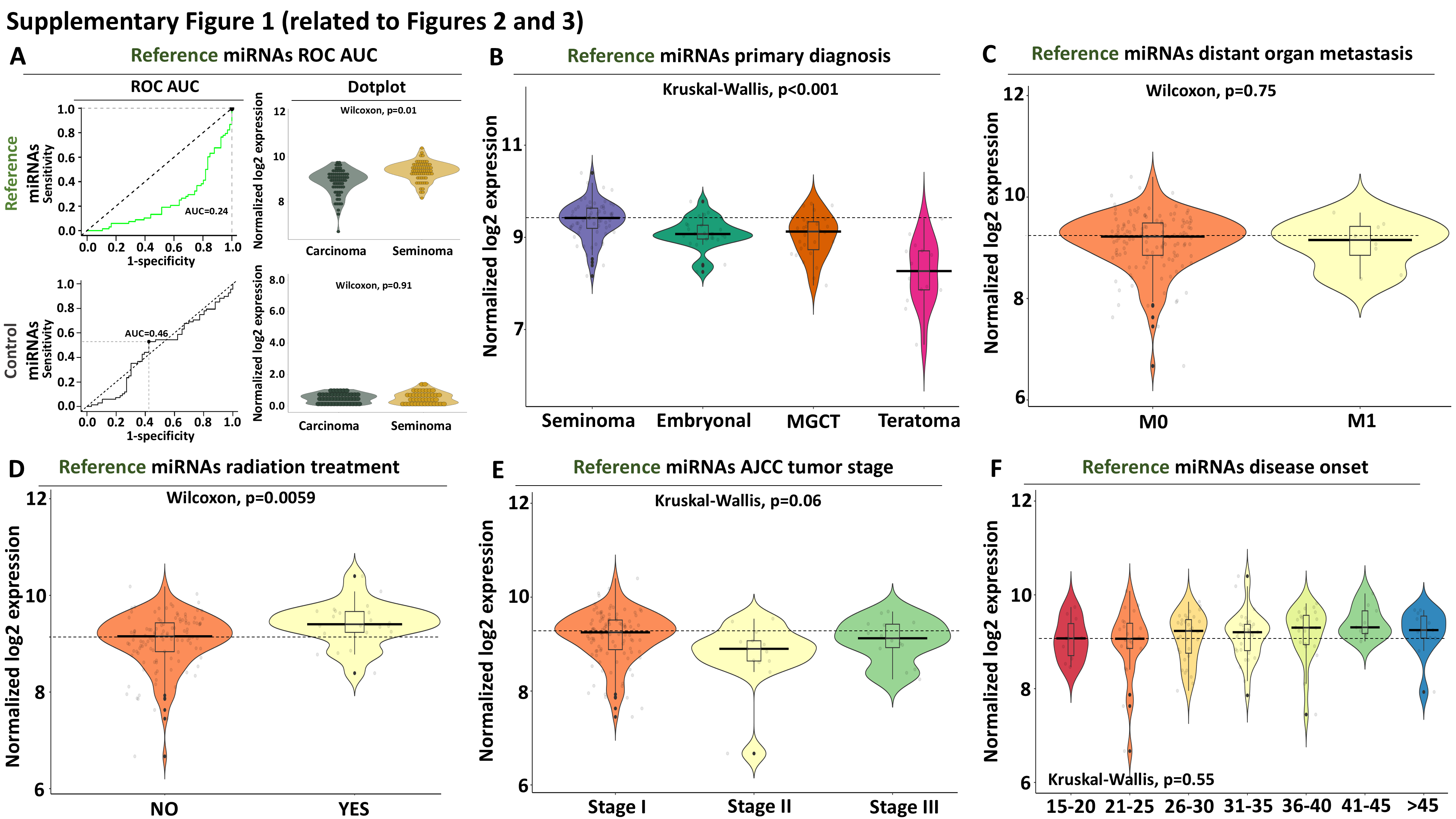

Supplement: Supplementary file 1 [file genes-15-01649-s001.zip › Supplementary Figure 1.TIF]

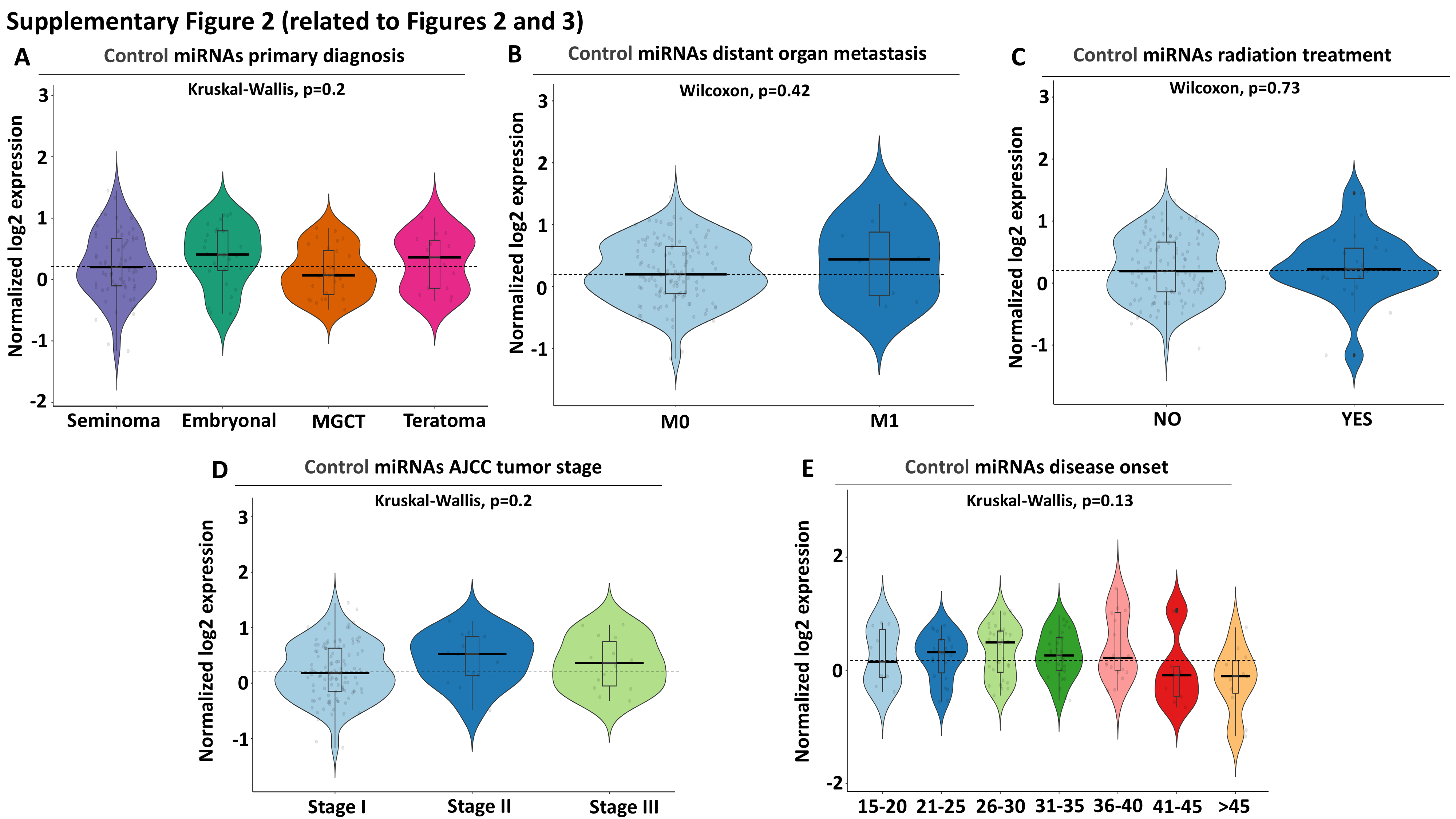

Supplement: Supplementary file 1 [file genes-15-01649-s001.zip › Supplementary Figure 2.TIF]

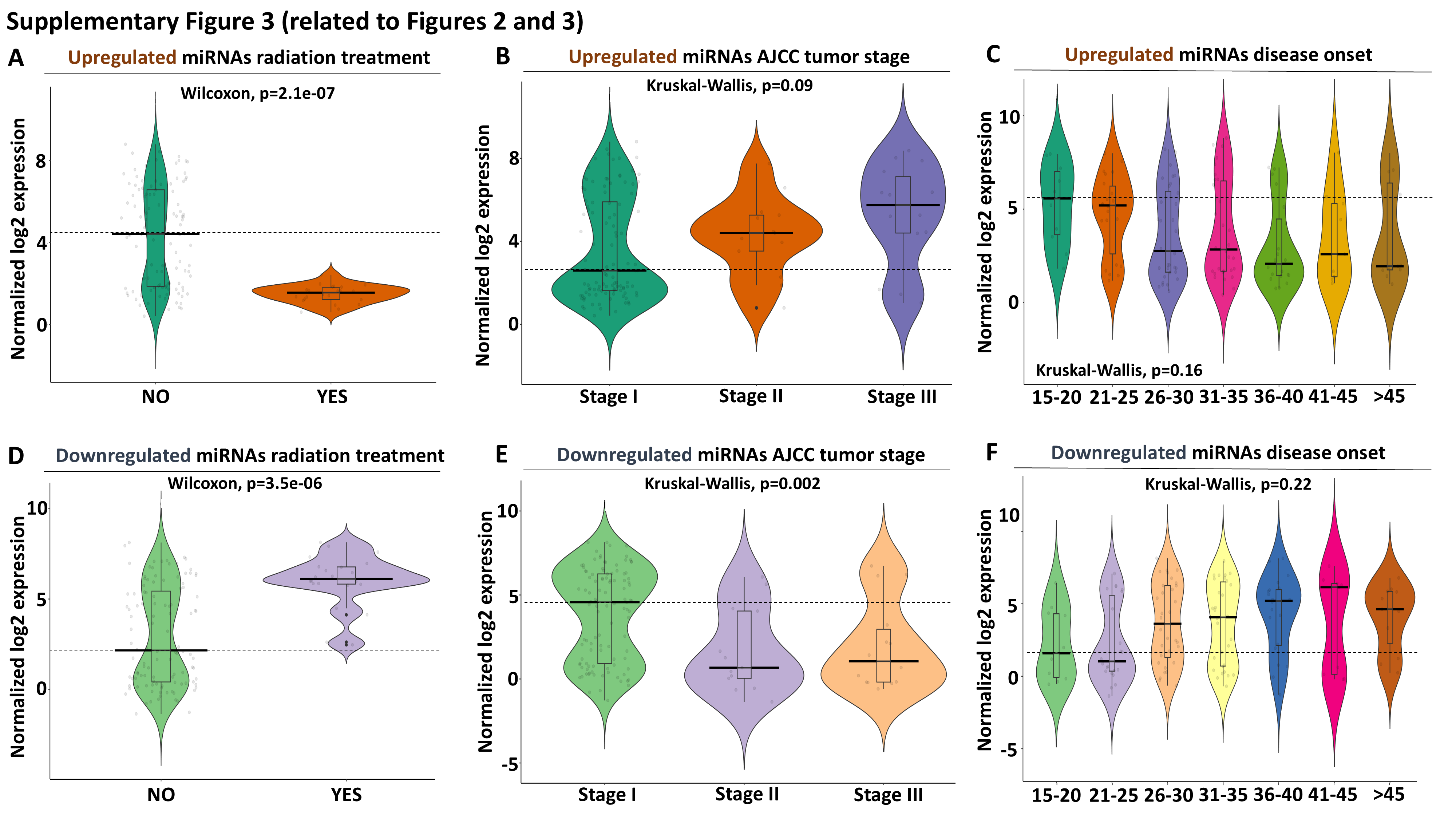

Supplement: Supplementary file 1 [file genes-15-01649-s001.zip › Supplementary Figure 3.TIF]
